# Risk factors of the progression to hypertension and characteristics of natural history during progression: A national cohort study

**DOI:** 10.1371/journal.pone.0230538

**Published:** 2020-03-17

**Authors:** Kwan Hong, Eun Sun Yu, Byung Chul Chun

**Affiliations:** 1 Department of Preventive Medicine, Korea University College of Medicine, Seoul, Korea; 2 Graduate School of Public Health, Korea University, Seoul, Korea; 3 National Health Insurance Service, Wonju-si, Korea; International University of Health and Welfare, School of Medicine, JAPAN

## Abstract

**Background:**

Although the high disease burden that results from cardiovascular complications of hypertension, factors related to the progression to hypertension in the normotensive population are not actively reported. The purpose of this study was to estimate the rate of the progression to hypertension and to reveal the associated risk factors.

**Methods:**

The study included normotensive participants from the National Health Insurance Service-National Health Screening Cohort, and contained a 10% sample of all adults who received a national health screening test in either 2002 or 2003. At the end of the study in 2015, the patients were divided into two groups based on whether or not they progressed to hypertension. Cox proportional hazard modeling was performed to identify risk factors for progression. Subgroup analysis using logistic regression was employed to reveal factors influencing the different natural history of the progression.

**Results:**

Among the 75,335 included participants, the progression rate to hypertension was 66.39% (50,013), with an adjusted incidence rate of 8.62 per 100 person-year in the aged 40–64 group and 12.68 in the aged 65 or above group. Age, BMI, hemoglobin, and family history of hypertension and other diseases were related to the progression. Among the progression group, 78.21% (39,116) participants skipped a pre-hypertensive status; this group consisted of older females with lower pulse pressure and more alcohol consumption compared to people who had pre-hypertensive status before the progression.

**Conclusion:**

Substantial risk factors for the progression to hypertension should be carefully managed even in normotensive participants who receive health screening tests.

## Introduction

Hypertension is the leading component of global disease burden and acts as a major cause of cardiovascular diseases [1]; a higher mortality in hypertensive population is well known in many countries through national level studies [2–4]. However, the incidence and prevalence of hypertension is difficult to measure naturally, given its asymptomatic nature [5]. Many studies that have aimed to determine the prevalence of hypertension by screening or survey have concluded that 20%–45% of the total population worldwide has hypertension [6–9]. However, the definition of hypertension changed in the 2017 American College of Cardiology/American Heart Association (ACC/AHA) hypertension guidelines [10], which caused confusion in the diagnosis and treatment of patients with stage 1 hypertension, previously referred to as pre-hypertension [11]. In this complex situation, and due to the dynamic nature of blood pressure, it is difficult to measure blood pressure in a consistent way [12] and classify the blood pressure status in the general population.

Nevertheless, classifying and revealing risk factors for the progression to hypertension is quite meaningful considering its contribution to health status. Since the new diagnostic criteria is not applied to all countries, there are currently a limited number of studies that are attempting to determine the risk factors of the progression to hypertension (stage 1 or 2 hypertension as referred to in the 2017 ACC/AHA hypertension guidelines) or comparing the groups who passed different steps of the natural history (those who skipped pre-hypertensive step or not). Indeed, only the natural history of the stage 1 hypertension group (previously called the pre-hypertensive group) was revealed in the majority of studies [13–17]. Therefore, this study aims to identify the incidence rate and risk factors for the progression from normotensive to hypertensive using the national cohort study. Additionally, we aimed to reveal factors associated with the different natural history of the progression group.

## Materials and methods

### Study population

**Fig 1:**
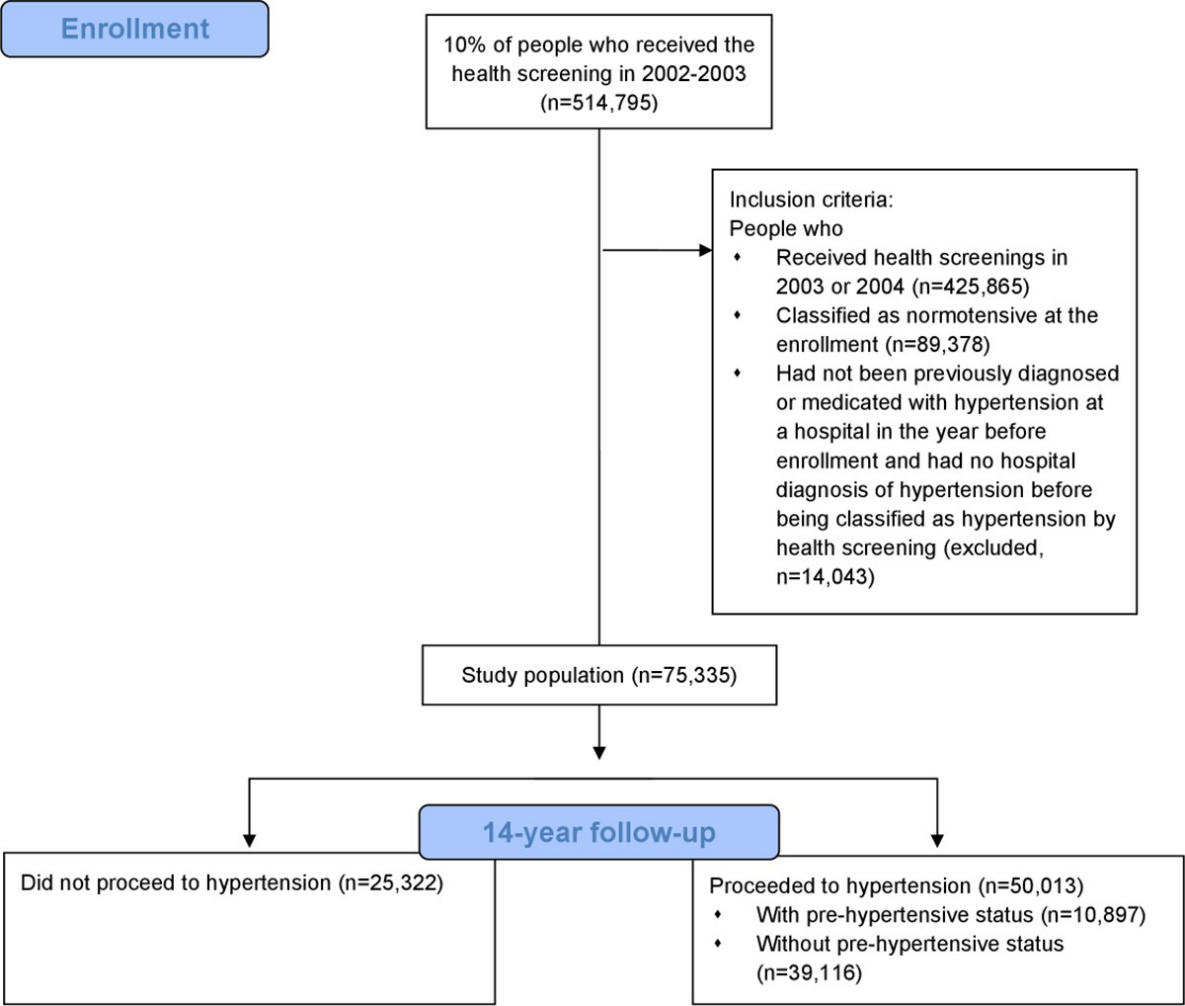
Flow chart of the study.

Data from The National Health Insurance Service-National Health Screening Cohort (NHIS-HEALS) in Korea were used in this study. Fig 1 shows overall population and procedure of this study. The data included 514,795 participants who were randomly sampled from 10% of the population who received the health screening in 2002 or 2003 and followed up until 2015 [18]. Given the sensitive nature of the data, requests to access the dataset from qualified researchers may be sent online to the National Health Insurance Sharing Service system in Korea [19]. For the re-analysis of this study, the codes for the analysis is available from the first author on request.

The health screening program in Korea provides health check-ups biannually to all subjects aged 40–79 years who are covered by the national health insurance. These data include all hospital data for which insurance was claimed. Among this population, we constructed our cohort as the following definition: People (1) who received health screenings in 2003 or 2004 and were classified as normotensive, (2) who had not been previously diagnosed with hypertension at a hospital in the year before enrollment, and (3) who had no hospital diagnosis of hypertension before being classified with hypertension by health screening. We followed the criteria from the 2017 ACC/AHA hypertension guideline for the classification of hypertension.

### Study procedure

Using the regular blood pressure measurement of participants by health screening tests, we classified their blood pressure status as normotensive (< 120/80 mmHg), pre-hypertensive (120–129/< 80 mmHg), stage 1 hypertensive (130–139/80–89 mmHg), and stage 2 hypertensive (≥ 140/90 mmHg) in each measurement in order to follow-up their natural history. Only normotensive participants at registration were included in analysis in this study. After 14 years of follow-up, the study population was grouped according to whether they had progressed to hypertension at the end. After estimating the incidence rate using the number of hypertensive patients and the total follow-up years of all participants, we extracted the possible risk factors for progression in each participant. Demographic factors such as age and sex, factors associated with health behaviors such as smoking status, alcohol consumption, and exercise status, past history of diabetes, family history of diseases including hypertension and stroke, and measured variables from health screening tests such as BMI, hemoglobin, total cholesterol, fasting glucose, and pulse pressure were included in the analysis. We conducted subgroup analysis in the progressed group in order to determine any difference in natural history between people who are classified as pre-hypertension during biannual follow-up and people who skipped this status. The former indicated a slower step-by-step progression to hypertension.

### Statistical analysis

To identify the risk factors for progression to hypertension, chi-square statistics for categorical variables and t-test for continuous variables were used for the selection of factors at the level of 0.1 by p-values, stratified by sex. The selected risk factors were included in a multivariable Cox proportional regression model using a stepwise selection method. The hazard ratios and 95% confidence intervals of each risk factor were presented, and the adequacy of the final model was checked by AIC (Akaike Information Criterion) statistics. The linearity and proportionality of the model was verified through the log-cumulative hazard plot and Shoenfeld residual [20]. Due to missing values of each of the factors, we evaluated the model stability by subtracting one variable each and comparing it with the total model. The crude and adjusted incidence rate was estimated additionally, stratified by age and sex.

The progression group was selected for the subgroup analysis. This group was further divided into two groups based on whether or not the participants were classified as pre-hypertensive before hypertension. Similarly, the difference in characteristics between the two groups was tested by chi-square statistics and t-tests and selected for multivariate analysis at the level of p-value < 0.1. Logistic regression analysis was used to reveal the adjusted effect of each risk factor and the odds ratio with 95% confidence interval are presented. AUC (Area Under the Curve) and the Hosmer-Lemeshow test were used to assess the fitness of the final model [21].

Additionally, Pearson correlation coefficients between continuous variables, including age, pulse pressure, BMI, total cholesterol, and fasting glucose, are estimated. All of them were under 0.3, indicating low correlation among these values. Statistical significance of both multivariate analyses was accepted with a p-value = 0.05, and all statistical analysis was conducted using SAS version 9.4 (SAS Institute Inc).

### Ethical approval

This project was approved by the institutional review board of Korea University (IRB: KUIRB-2018-0064-01). Informed consent was waived because personal information was deleted from the dataset.

## Results

Among the 425,865 individuals who received health screening tests in 2003 or 2004, 89,378 (20.99%) were normotensive and 75,335 participants who met the inclusion criteria were included in the study. At the end of the follow-up in 2015, 50,013 (66.39%) participants proceeded to hypertension. The crude and adjusted incidence rate of hypertension is presented in Table 1; the incidence rate was 8.93 per 100 person-year in individuals younger than 65 years old and 13.18 per 100 person-year in individuals older than 65 years old. The incidence rate ratio between the two age groups was 1.47 (95% confidence interval: 1.41–1.53).

**Table 1:** Crude and adjusted^a^ incidence rate (per 100 person-year) and 95% confidence intervals of hypertension stratified by age and sex.

	≤ 65 years old			> 65 years old		
	Male	Female	Total	Male	Female	Total
Crude Incidence rate	11.14 (10.98, 11.31)	7.61 (7.51, 7.72)	8.93 (8.84, 9.02)	13.47 (12.80, 14.17)	12.93 (12.32, 13.57)	13.18 (12.72, 13.65)
Adjusted Incidence rate	10.14 (9.65, 10.66)	7.32 (6.96, 7.70)	8.62 (8.22, 9.04)	13.15 (12.22, 14.15)	12.22 (11.36, 13.15)	12.68 (11.91, 13.49)

^a^Adjusted for health behaviors, family history of diseases, past history of diabetes, fasting glucose, pulse pressure, hemoglobin, and BMI.

### Risk factors for the progression to hypertension

The baseline characteristics and the univariate analysis of the study population are described in Table 2. The mean age of the study population was 50.25±8.04 (mean±standard deviation) years old, and the people who proceed to hypertension were slightly older (50.57±8.14 compared to 49.63±7.80). The proportion of males was significantly higher in the progression group, accounting for 46.58% compared to 34.42% of the non-progressing group. Additionally, almost all of the remaining factors were different in either their mean or percentage in the two groups. The mean blood pressure and the mean pulse pressure were higher in the progressing group, 107/67 (pulse pressure 39.97) mmHg in the progressing group and 105/65 (pulse pressure 39.41) in the non-progressing group. The BMI was also higher in the progressing group, with a mean BMI of 23.16±2.70 kg/m^2^ in the progressing group compared to 22.36±2.55 kg/m^2^ in the non-progressing group. The total cholesterol, hemoglobin, and fasting glucose were also higher in the progressing group (p < 0.05). The past history of diabetes was not significantly different in the two groups, but more people tended to have diabetes in the progressing group (1.99% compared to 1.84%). In terms of the family history of diseases, hypertension and stroke were positively related with the progression to hypertension, while other histories, such as diabetes, cancer, and liver disease, related negatively. Current smokers progressed to hypertension more than non-smokers (21.35% compared to 17.24%).

**Table 2:** Descriptive characteristics^a^ and univariate analysis of the study population.

	No progression to hypertension (n = 25,322)	Progression to hypertension (n = 50,013)	P-value
Age	49.63 ± 7.80	50.57 ± 8.14	< 0.01
Sex (male)	8,717 (34.42)	23,296 (46.58)	< 0.01
Systolic BP (mmHg)	104.70 ± 7.80	107.20 ± 6.96	< 0.01
Diastolic BP (mmHg)	65.26 ± 6.30	67.20 ± 5.77	< 0.01
Pulse pressure (mmHg)	39.41 ± 6.79	39.97 ± 6.28	< 0.01
BMI (kg/m^2^)	22.36 ± 2.55	23.16 ± 2.70	< 0.01
Total cholesterol (mg/dL)	191.10 ± 35.16	193.90 ± 35.77	< 0.01
Hemoglobin (g/dL)	13.26 ± 1.49	13.62 ± 1.53	< 0.01
Fasting glucose (mg/dL)	91.27 ± 23.26	92.40 ± 25.54	< 0.01
Past history of diabetes	467 (1.84)	993 (1.99)	0.18
Family history of			
Hypertension	1,420 (6.18)	2,967 (6.56)	0.06
Stroke	1,165 (5.08)	2,367 (5.24)	0.36
Diabetes	1,806 (7.85)	3,159 (6.98)	< 0.01
Cancer	4,050 (17.46)	7,027 (15.42)	< 0.01
Liver disease	939 (4.10)	1,502 (3.33)	< 0.01
Current smoker	4,208 (17.24)	10,293 (21.35)	< 0.01
Alcohol consumption (≥ 3 times/week)	1,335 (5.37)	3,958 (8.06)	< 0.01
Physical exercise (≥ 3 times/week)	5,016 (20.30)	9,560 (19.59)	0.02

^a^Continuous variables are presented as mean ± SD (standard deviation) and categorical variables are presented as number (%).

Since the sex of participants were strongly related with health behaviors such as smoking and alcohol consumption, we stratified the study population by sex and selected the possible risk factors, age, BMI, hemoglobin, family history of hypertension/cancer/liver disease and alcohol consumption in males and age, pulse pressure, BMI, hemoglobin, fasting glucose, family history of diabetes/cancer/liver disease, smoking status and physical exercise in females, in each stratum by univariate analysis. After the selection, the adjusted hazard ratio and 95% confidence intervals are presented in Table 3, using Cox proportional regression modeling by the stepwise selection method, considering the time-dependent effect of age. In males, age, BMI, hemoglobin, family history of hypertension, and alcohol consumption positively influenced the progression to hypertension (adjusted hazard ratios: 1.013, 1.051, 1.019, 1.106, and 1.130). Family history of cancer or liver disease negatively related to the progression with adjusted hazard ratios of 0.928 and 0.911, respectively. Similarly, in females, age, BMI, and hemoglobin were positively related (adjusted hazard ratios: 1.033, 1.043, and 1.028), but family history of hypertension and alcohol consumption were not included in the final model. In addition, pulse pressure and fasting glucose affected the progression in females (adjusted hazard ratios: 1.005 and 1.001). Family history of diabetes, cancer, and liver disease were negatively related with the progression in females, with measured hazard ratios of 0.948, 0.920, and 0.838, respectively. In females, current smoking status and physical exercise lowered the progression to hypertension, with adjusted hazard ratios of 0.559 and 0.928, respectively.

**Table 3:** Adjusted hazard ratios^a^ of the progression to hypertension by sex.

	Male (n = 32,013)	Female (n = 43,322)
Age	1.013 (1.009, 1.017)	1.033 (1.030, 1.036)
Pulse pressure (mmHg)	-	1.005 (1.003, 1.007)
BMI (kg/m^2^)	1.051 (1.046, 1.057)	1.043 (1.034, 1.052)
Hemoglobin (g/dL)	1.019 (1.006, 1.003)	1.028 (1.016, 1.039)
Fasting glucose (mg/dL)	-	1.001 (1.000, 1.001)
Family history of		
Hypertension	1.106 (1.039, 1.178)	-
Diabetes	-	0.948 (0.901, 0.996)
Cancer	0.928 (0.891, 0.966)	0.920 (0.887, 0.954)
Liver disease	0.911 (0.841, 0.986)	0.838 (0.776, 0.906)
Current smoker	-	0.559 (0.463, 0.674)
Alcohol consumption (≥ 3 times/week)	1.130 (1.087, 1.174)	-
Physical exercise (≥ 3 times/week)	-	0.928 (0.898, 0.960)

Stepwise selection of significant variables, p-value under 0.05.

The difference of AIC (Akaike Information Criterion) statistics of the final model and the null model was 521.07 in males and 1738.47 in females.

^a^Hazard ratios of the progression to hypertension are presented with 95% confidence intervals.

### People who skipped pre-hypertensive status and proceeded to hypertension directly

Table 4 shows the subgroup analysis of the progression group, comparing people with and without pre-hypertensive status during biannual follow-up; 78.21% (39,116) of the progression group skipped pre-hypertensive status. The adjusted odds ratios were measured by logistic regression analysis with the stepwise selection method after selecting the included variables by univariate analysis. The mean age was higher in the progression group (50.85 years old compared to 49.58 years old), with an adjusted odds ratio of 1.020 (95% confidence interval: 1.017–1.023). Males, people with higher pulse pressure, and lower alcohol consumption had increased pre-hypertensive status (adjusted odds ratios: 0.912, 0.977, and 0.805, respectively).

**Table 4:** Subgroup analysis of the progression group: Differences between individuals with and without a pre-hypertension status^a^.

	Pre-hypertensive status (n = 10,897)	No pre-hypertensive status (n = 39,116)	Adjusted odds ratio^b^	95% confidence intervals
Age	49.58 ± 7.59	50.85 ± 8.27	1.020	(1.017, 1.023)
Sex (male)	5,190 (47.63)	18,106 (46.29)	0.912	(0.871, 0.956)
Pulse pressure (mmHg)	40.65 ± 6.30	39.78 ± 6.26	0.977	(0.973, 0.98)
Alcohol consumption (≥ 3 times/week)	761 (7.11)	3,197 (8.32)	0.805	(0.735, 0.881)

Stepwise selection of significant variables, p-value under 0.05.

The overall AUC (Area Under Curve) was 0.56 (95% confidence interval = 0.55 to 0.57).

Hosmer-Lemeshow goodness-of-fit test: chi-square = 5.82, df = 8 and p-value = 0.67.

^a^Continuous variables are presented as mean ± SD (standard deviation) and categorical variables are presented as number (%).

^b^Adjusted odds ratios were measured by logistic regression analysis with stepwise selection.

The Cox proportional hazard model we used in Table 3 was selected considering minimal AIC statistics after stepwise variable selection. The difference of AIC statistics of the final model and the null model was 521.07 in males and 1738.47 in females. After testing the linearity and proportionality, time-dependent interaction terms of age and pulse pressure in males and age, smoking status, BMI in females were included in the final model. The logistic regression model used in Table 4 used stepwise selection method to select variables and tested for goodness-of-fit by Hosmer-Lemeshow test, resulted chi-square = 5.82, df = 8 and p-value = 0.67. The overall AUC was 0.56 (95% confidence interval = 0.55–0.57).

## Discussion

In this national cohort-based study, the progression rate from normal to hypertension was 66.39%, with an adjusted incidence rate of 8.62 per 100 person-year in middle-aged people and 12.68 per 100 person-year in elderly people; this was a much larger proportion than previous studies have demonstrated [5, 9, 15, 22], including the population study in Japan, which reported a total of 40.7% of progression to hypertension at the 12-year follow up [17]. To the best of our knowledge, this is the first study to report the progression rate and risk factors of the progression to hypertension in Korea. The results of the current study indicate a substantial risk of an increased hypertensive population. Since there is strong evidence of the cardiovascular risk of the population who are diagnosed with hypertension by the 2017 ACC/AHA diagnostic criteria [23], the management of hypertension may also change to include the larger population at risk.

Identifying risk factors of the progression to hypertension in the normotensive population is more informative than simply comparing hypertensive and normotensive groups because it can actually reflect what risk factors are to be found and managed through screening programs. Age, BMI, and hemoglobin level, which showed an increase in progression in both males and females, can be applied as the observed indicator of the progression, even in the normotensive period. In agreement with our results, there is considerable evidence of these factors as a possible risk factors of hypertension [16, 24–28].

Since this study included only normotensive population at the enrollment, there were minimal effect of baseline blood pressure in results, decreasing only 1.9 AIC statistics with inclusion. Instead of adjusting baseline blood pressure, we used pulse pressure as the risk factor and showed a little increase of the progression to hypertension with adjusted hazard ratio 1.005 in females. There were two factors that showed a positive relation with progression in females: pulse pressure and fasting glucose. Although these factors are known to increase progressively with age [29, 30], this result is important since adjustments were made to account for the time-dependent age effect. Although this difference has not been previously discussed, it indicates that females may be more vulnerable to fasting glucose level or pulse pressure.

In addition, exercise habits seemed to only be protective in females; however, the questionnaire only asked for the frequency of exercise and did not consider the intensity or time. Therefore, considering previous studies that have revealed the effect of exercise in the protection from hypertension [28, 31, 32], this result should be interpreted with caution. Alcohol consumption is widely known as a risk factor for hypertension [33, 34]; the results of the current study demonstrated an increased risk of hypertension in males with high alcohol consumption, but the same was not true for females. However, this may be because only 1.61% of females are classified as heavy alcohol consumers in our population. Surprisingly, in females, smoking showed an inverse relationship with the progression to hypertension. Indeed the potential relationship of smoking and hypertension remains controversial [35, 36], and further studies need to be conducted with a focus on the pathophysiology.

Family history of hypertension did increase the progression in males, showing that genetic predisposition can still be considered as a major risk factor [37, 38]. However, the family history of other diseases showed negative relationship with the progression; this infers that diseases such as diabetes or cancer can affect the health behaviors of individuals, leading to the control of blood pressure.

Subgroup analysis showed the difference between people who had pre-hypertensive status or not in the study period. In the group that skipped pre-hypertensive status, the rate of progression might be faster than the other group, or their health behavior might not be good enough to receive biannual health screening. Although there may be misclassification due to the latter, the results can have sufficient value since this was the first attempt to compare two groups with different natural histories of hypertension. After considering interaction and the effect of covariates, age, sex, pulse pressure, and alcohol consumption remained possible risk factors for directly proceeding to hypertension and skipping pre-hypertensive status. In other words, females with increase alcohol consumption, lower pulse pressure, and older age were at greater risk of skipping pre-hypertensive status.

There are several limitations to this study. Firstly, this study only included data from the national health screening result and hospital data, so other known risk factors, such as diet [39–41], cannot be considered; as an alternative to this, BMI and total cholesterol were included in the analysis. Also, the national health screening only included exercise pattern of participants as the frequency. The lack of information about the daily amount of physical activity might have affected the study result since there is the intervention study that revealed the protective effect of regular aerobic exercise. Lastly, some factors in the health screening test, such as family history or health behavior, were self-reported, which may be inaccurate and also lead to a higher missing rate (up to 9.7%). However, after subtracting each variable in the analysis in order to determine the potential bias, there were no change in the direction or significance of the final model.

## Conclusions

This large national cohort-based study revealed several potential risk factors of the progression to hypertension, including family history, health behavior, and objective values such as BMI and hemoglobin. Through this result, medical services can not only find out high-risk population for the progression to hypertension in pre-hypertensive people, but also manage them focusing on modifiable factors such as BMI. Furthermore, a discussion of the progression group comparing different natural history may help to develop a more efficient management and screening system for hypertension.
